# Long-term oral administration of naringenin counteracts aging-related retinal degeneration *via* regulation of mitochondrial dynamics and autophagy

**DOI:** 10.3389/fphar.2022.919905

**Published:** 2022-07-14

**Authors:** Guiping Chen, Ling Zeng, Feng Yan, Jinlong Liu, Mengqi Qin, Feifei Wang, Xu Zhang

**Affiliations:** ^1^ Affiliated Eye Hospital of Nanchang University, Jiangxi Clinical Research Center of Ophthalmic Disease, Jiangxi Provincial Key Laboratory for Ophthalmology, Nanchang, JX, China; ^2^ School of Pharmacy, Nanchang University, Nanchang, JX, China

**Keywords:** naringenin, aging eye, visual function, mitochondria dynamics, autophagy

## Abstract

Aging-related retinal degeneration can manifest as decreased visual function due to damage to retinal structures and dysfunction in retinal homeostasis. Naringenin, a flavonoid, has beneficial effects in preventing cellular aging, preserving the functionality of photoreceptors, and slowing down visual function loss. However, the role and potential mechanism of naringenin in the aging mouse retina require further investigation. In this study, we evaluated the effects of naringenin on the aging eye using electroretinogram (ERG) and hematoxylin and eosin staining and explored its potential mechanism by western blotting. ERG showed that naringenin increased the amplitude of the a- and b-waves of scotopic 3.0, 10.0, and the a-wave amplitude of photopic 3.0 in the aging mouse retina. Furthermore, administration of naringenin prevented aging-induced retinal degeneration in the total retina, ganglion cell, inner plexiform layer, inner nuclear layer, and outer nuclear layer. The expression of mitochondrial fusion protein two was increased, OPA1 protein expression and the ratio of L-OPA1/S-OPA1 were unchanged, and dynamin-related protein one was decreased in the 12-month-old mice treated with naringenin compared with the 12-month-old mice treated with vehicle. Furthermore, the downregulation of age-related alterations in autophagy was significantly rescued in the aging mice by treatment with naringenin. Taken together, these results suggest that the oral administration of naringenin improves visual function, retinal structure, mitochondrial dynamics, and autophagy in the aging mouse retinas. Naringenin may be a potential dietary supplement for the prevention or treatment of aging-related retinal degeneration.

## 1 Introduction

An aging eye is characterized by a gradual loss of visual function, which seriously affects quality of life (Lin et al., 2016). Aging of the eye that occurs in the retina can lead to irreversible blindness (Chen et al., 2019). However, there is a lack of safe and effective dietary supplements to counteract aging-related retinal alterations. Natural products have special resource advantage and with few side effect. Compelling evidence suggests the effective potential of natural product and phytocompounds to slow aging and extend lifespan (Ding et al., 2017; Sharma and Martins, 2020). Naringenin, a common ingredient in the human diet, is derived from grapefruit and citrus fruit. It has anti-inflammatory, antioxidant, anti-aging, and neuroprotective properties (Nouri et al., 2019; Wang et al., 2019). In addition, naringenin has beneficial effects in preventing cellular aging, and preserving the functionality of photoreceptors, and slowing down visual function loss (Ghofrani et al., 2015). The long-term oral administration of naringenin preserves retinal function by increasing photopic b-wave amplitude (Piano et al., 2021). Naringenin eye drops protect retinal function and morphology in NaIO_3_-induced retinopathy (Chen et al., 2021). These findings suggest that naringenin may be valuable for treating aging eyes. Nevertheless, its effect on the aging eye and the mechanism by which it does require further investigation.

Many aging-related eye diseases, including glaucoma and age-related macular degeneration (AMD) are associated with mitochondrial dysfunction (Eells, 2019). Dysfunction of mitochondrial dynamics may be one of the internal causes of mitochondrial dysfunction, leading to oxidative stress responses and cell death during aging (Li et al., 2018). Mitochondrial fusion is joining multiple mitochondria and is mediated by optic atrophy 1 (OPA1) and mitochondrial fusion proteins one and 2 (MFN1 and MFN2). Loss of long-OPA1 (L-OPA1) with concomitant accumulation of short-OPA1 (S-OPA1) induces mitochondrial fragmentation (Jang and Javadov, 2020). Mitochondrial fission divides a single large mitochondrion into multiple smaller mitochondria. This division is mediated by dynamin-related protein 1 (DRP1) and mitochondrial fission protein 1 (Lee and Yoon, 2016). However, whether naringenin can improve mitochondrial dysfunction during retinal aging remains unclear.

Autophagy is a process in which intracellular components, such as damaged or superfluous organelles or aggregated proteins, are engulfed by autophagosomes and degraded within lysosomes. Mitochondrial fission allows the selective separation of damaged mitochondria, which can then be eliminated by autophagy (Tagaya and Arasaki, 2017). Microtubule-associated protein one light chain 3 (LC3) is a soluble protein with a molecular mass of approximately 17 kDa that is distributed ubiquitously in mammalian tissues and cultured cells. LC3B is involved in autophagosome formation, and the conversion of LC3-I to LC3-II is a characteristic signature of autophagic membranes (Dikic and Elazar, 2018). P62 is an autophagy adaptor protein that promotes the formation and degradation of ubiquitinated aggregates (Aparicio et al., 2019). Autophagy is regulated by various signaling pathways. Adenosine monophosphate-activated protein kinase (AMPK), a metabolic sensor of energy balance, plays an important role in the regulation of energy homeostasis and induces autophagy by downregulating phosphorylation of rapamycin (mTOR) (Chen et al., 2018). A decline in autophagy activity with age has been observed in the retina and is believed to cause aging-related diseases (Liu and Levine, 2015). Therefore, regulating autophagy and mitochondrial dynamic balance may be a future treatment strategy for age-related eye diseases, such as glaucoma and AMD (Wang et al., 2015). In this study, we explored the effect of naringenin on autophagy induced by retinal aging.

Given the wide range of the pharmacological properties of naringenin, we investigated its effects on aging-mediated retinal structure and function degeneration, and elucidated the underlying molecular mechanisms.

## 2 Methods

### 2.1 Animals and drug treatment

This study strictly adhered to the Association for Research in Vision and Ophthalmology Statement for the Use of Animals in Ophthalmic and Vision Research and was approved and monitored by the Institutional Animal Care and Use Committee of Nanchang University, China (Permit Number: SCXK (Xiang) 2019–0,013). Male healthy C57BL/6J mice (7 weeks) were provided by Changsha Tianqin Biotechnology Co., Ltd. Mice with eye disease were excluded. The animals were maintained under a 12 h light/dark cycle in a temperature and humidity-controlled room. Mice were provided with standard chow and sterile water. When the animals adapted to the laboratory for a week, they were randomly placed in groups of four per cage. To evaluate the effects of long-term treatment, 2-month-old male mice were divided into four groups (*n* = 20; 8 weeks old, 20–23 g): 1) 2 M (normal 2-month-old mice); 2) 12 M (normal 12-months-old mice); 3) 12 M + vehicle [0.5% carboxymethylcellulose sodium (CMC-Na) administered orally to 2-month-old mice for 10 months]; 4) 12 M + naringenin (2-month-old male mice were treated with naringenin (≥98%, Cat no. N107346, Aladdin Biotechnology, Shanghai, China) by oral gavage at a dose of 100 mg/kg/day (dissolved in CMC-Na solution) for 10 months). Each group consisted of five mice (*n* = 5). Naringenin was administered orally through oral gavage. To alleviate the pain caused by intragastric administration, mice were anesthetized with isoflurane during continuous intragastric administration (Jones et al., 2016).

### 2.2 Electroretinogram

The mice were tested with ERG to assess retinal function after treatment with naringenin. The mice were exposed to dim red light following dark overnight adaptation (>12 h) and anesthetized with isoflurane and chloral hydrate at 3.6% (10 ml/kg) to maintain anesthesia. Tropicamide (1%) was used to dilate pupils. Mice temperature was maintained at 37°C with a heating pad. Stainless steel wire loops (0.1 mm diameter) were placed on the center of the cornea with 1% methylcellulose to prevent corneal dehydration. A reference electrode was placed at the midpoint of the line between the eye and ear, and a grounding electrode was placed near the tail. Each mice group was subjected to the guidelines of the International Society for Clinical Electrophysiology of Vision, including the scotopic 0.01 ERG test (rod response) elicited by white light flashes at an intensity of 0.003 phot cd·s/m^2^, the scotopic 3.0 ERG test (cone and rod response) elicited by white light flashes at an intensity of 3.0 phot cd·s/m^2^, the scotopic 3.0 oscillatory potential ERG test simultaneously elicited by white light flashes at an intensity of 20.0 phot cd·s/m^2^, and the photopic 3.0 ERG test (cone response) elicited with white flashes under white background elicited by white light flashes at an intensity of 2.8 phot cd·s/m^2^ under white background light at 29.0 phot cd/m^2^ after 10 min of light adaptation. The amplitude of the a-wave was measured from the baseline to the trough, whereas that of the b-wave was measured from the maximum of the a-wave trough to the peak of the b-wave. The amplitude of the OS2-wave was measured from the maximum of the N2-wave trough to the peak of the P2-wave.

### 2.3 Measurement of biochemical and physiological indices

At the end of the naringenin treatment, we first measured the body weight and body length of the mice, and then blood was collected from the eyeballs (Zheng et al., 2019). The blood was made to stand at room temperature for 1h, then centrifuged at 1,000 g and 4°C for 10 min. The clear supernatant (plasma) was collected and used immediately to determine the levels of total cholesterol, triglyceride, high-density lipoprotein, low-density lipoprotein, fasting blood glucose, serum albumin, and alanine transporter.

### 2.4 Hematoxylin and eosin staining

The mice were euthanized by an intraperitoneal injection of 2% sodium pentobarbital (100 mg/kg), and the heartbeat, breathing and corneal reflex were observed. After verification of animal death through a lack of heartbeat, breathing or respiration, the eyeballs were removed for fixation and dehydration and embedded in a paraffin block. After the paraffin sections were dewaxed, the retinal sections were stained with H&E to observe retinal morphology. The retinal layer thickness was measured using Image-Pro Plus 6.0. The slices were selected at a distance of 1–2 mm from the optic disc under a light microscope.

### 2.5 Western blotting

Fresh retinas were lysed separately in a radioimmunoprecipitation assay lysis buffer containing a phosphatase inhibitor. After sonication, the supernatant was collected *via* centrifugation. The protein concentration was determined using a bicinchoninic acid assay (Vazyme Biotech Co., Ltd., Nanjing, China). The protein supernatant (20 μg) was analyzed by sodium dodecyl sulfate-polyacrylamide gel electrophoresis on a 10% gel and transferred by electroblotting to a polyvinylidene difluoride membrane (PVDF, Millipore, United Kingdom). The membrane was then blocked in Tris-buffered saline containing 0.1% Tween-20 and 5% skim milk for 1 h at room temperature. Membranes were incubated with primary antibodies, followed by incubation with peroxidase-conjugated secondary antibodies, and the protein bands were exposed to the EasySee Western Blot Kit and specific antibodies, including OPA1 (BD, 612606, 1:1000), MFN2 (Abcam, ab56889, 1:1000), DRP1 (CST, #8570S, 1:1000), P-AMPK (CST, #2535S, 1:1000), AMPK (CST, #5832, 1:1000), P-mTOR (CST, #5536, 1:1000), mTOR (CST, #2983, 1:1000), LC3B (CST, #2775, 1:1000), P62 (CST, #39749, 1:1000), β-tubulin (CST, #2128, 1:1000), and GAPDH (CST, #5174, 1:1000). Digital images were collected, and quantitative analysis was performed using ImageJ software.

### 2.6 Statistical analysis

All the quantified data represented the average of at least three samples. GraphPad Prism software (version 6.0) was used for statistical analysis. All data were analyzed using the unpaired Student’s t-test or one-way ANOVA, followed by the post hoc Tukey test. Unpaired Student’s t-test was used to compare the two groups. If more than two groups were analyzed, one-way ANOVA followed by the post hoc Tukey test was used. All data were expressed as the mean ± SEM. Statistical significance was considered at *p* < 0.05.

## 3 Results

### 3.1 Naringenin preserved retinal function in aging mice

**FIGURE 1:**
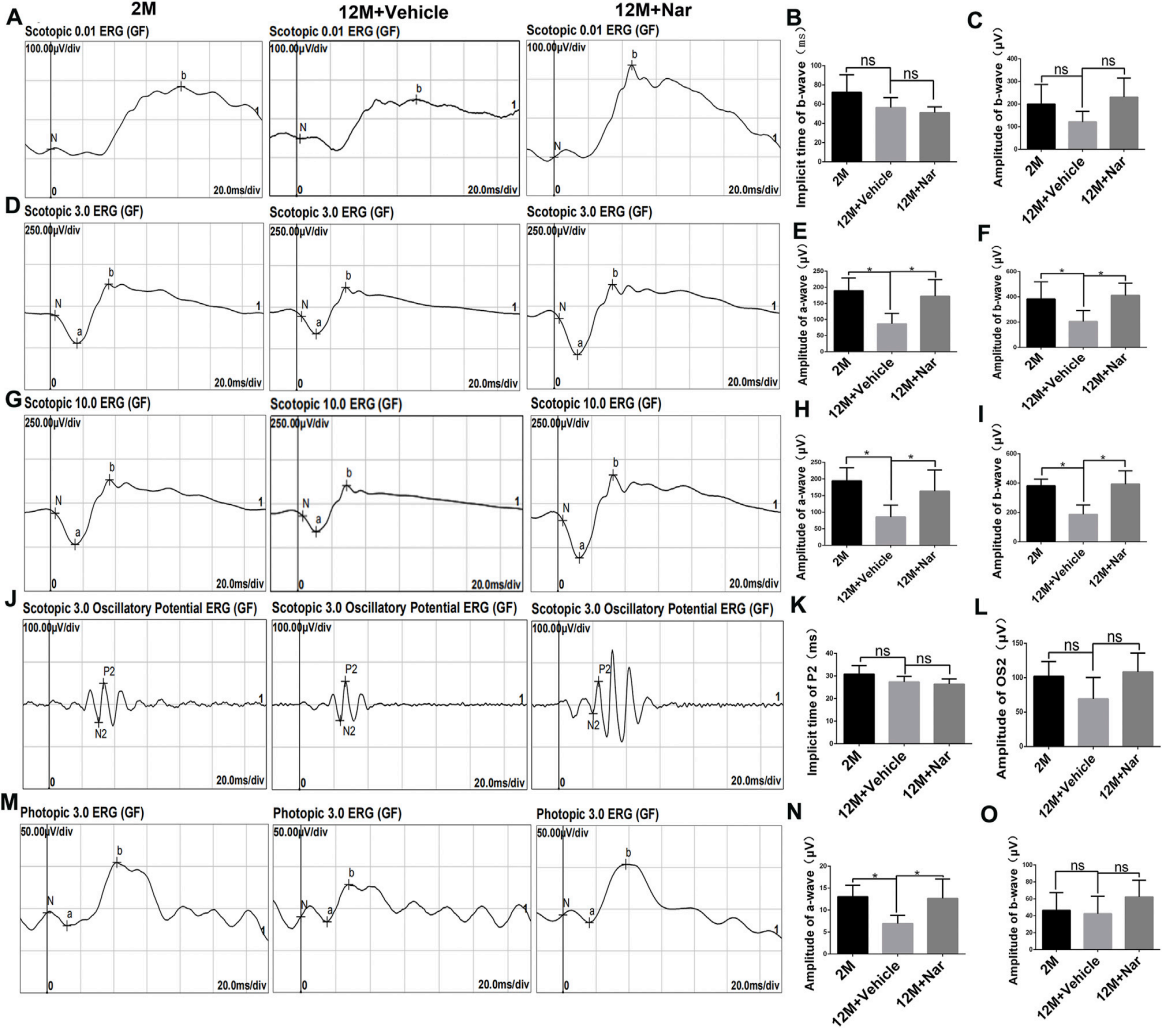
Naringenin (Nar) preserved retinal function of aging mice. **(A)** Representative scotopic 0.01 electroretinogram (ERG) waveforms, **(B)** the implicit time of b-wave, and **(C)** the amplitude of b-wave. **(D)** Representative scotopic 3.0 ERG (the combined rod-cone response [standard flash]) waveforms, the amplitudes of **(E)** a-wave and **(F)** b-wave. **(G)** Representative scotopic 10.0 ERG waveforms, the amplitudes of **(H)** a-wave and **(I)** b-wave. **(J)** Representative scotopic 3.0 oscillatory potential ERG, **(K)** the implicit time of P2-wave, and **(L)** the amplitude of OS2-wave. **(M)** Representative photopic 3.0 ERG waveforms, the amplitudes of **(N)** a-wave and **(O)** b-wave. 2 M: normal 2-month-old mice, 12 M: normal 12-month-old mice, 12 M + Vehicle: 0.5% CMC-Na treated 12-month-old mice, 12 M + Naringenin: Naringenin (100 mg/kg/day) treated 12-months-old mice. The results are expressed as the mean values ± SEM, *n* = 6. The results are expressed as the mean values ± SEM, *n* = 6. One-way ANOVA, post hoc Tukey’s test (*p < 0.05).

To explore the effect of naringenin on retinal electrophysiology in the aging mice, we conducted ERG in 2-month-old mice, 12-month-old mice treated with vehicle, and 12-month-old mice treated with naringenin. ERG showed no significant difference in implicit time and amplitude in scotopic 0.01 (rod response) (Figures 1A–C) in 12-month-old mice treated with naringenin compared to 12-month-old mice treated with the vehicle. The amplitudes of the a-wave and b-wave in the 12-month-old mice treated with naringenin were increased in scotopic 3.0 (the combined rod-cone response [standard flash]) and 10.0 ERG compared to the 12-month-old mice treated with vehicle (*p < 0.05; Figures 1D–I). In scotopic 3.0, the oscillatory potential, the amplitude of the OS2-wave, and the implicit time P2-wave were not significantly different in the 12-month-old mice treated with naringenin compared with the 12-month-old mice treated with vehicle (Figures 1J–L). In photopic 3.0 ERG (cone response), the implicit time of b-wave in all groups showed no differences; interestingly, a significant recovery of a-wave amplitude was observed in the 12-month-old mice treated with naringenin compared with the 12-month-old mice treated with vehicle (*p < 0.05; Figures 1M–O).

### 3.2 The effect of naringenin on biochemical parameters in mice

Long-term oral administration of naringenin may cause changes in physiological and metabolic parameters. To evaluate aging-related changes, we first measured body weight, body length, and glucose and lipid levels (Table 1). We observed that the bodyweight of the aging mice was significantly increased; however, it was reversed by treatment with naringenin (^#^ *p* < 0.05). This result was similar to that obtained from the long-term intake of nicotinamide mononucleotide (NMN) in inhibiting aging-related weight gain (Mills et al., 2016). Other biochemical parameters were not statistically different.

**TABLE 1:** The effect of Nar on biochemical and physiological indices in mice.Shown here are the means ± SEM of the data collected from three to five animals in each experimental group.

Parameter	2M	12M	12M + Vehicle	12M + Naringenin
Body weight(g)^a^	22.02 ± 0.35	31.2 ± 1.12^**^	29.66 ± 0.53	25.98 ± 1.3^#^
Body length (cm)^a^	8.33 ± 0.33	9.18 ± 0.6	8.69 ± 0.19	9.42 ± 0.76
T-CHO(mmol/L)^b^	2.23 ± 0.2	2.17 ± 0.16	2.41 ± 0.32	2.35 ± 0.73
TG(mmol/L)^b^	0.94 ± 0.21	0.99 ± 0.07	0.82 ± 0.19	0.41 ± 0.15
HDL(mmol/L)^b^	1.7 ± 0.18	1.52 ± 0.13	1.35 ± 0.45	1.57 ± 0.94
LDL(mmol/L)^b^	0.31 ± 0.08	0.22 ± 0.1	0.40 ± 0.55	0.51 ± 0.1
FBG(mmol/L)^b^	9.44 ± 0.35	12.20 ± 1.69	11.57 ± 3.04	8.24 ± 0.84
SA(g/L)^b^	31.5 ± 2.0	30.4 ± 0.8	28.90 ± 1.07	26.95 ± 2.2
AT(U/L)^b^	29.90 ± 3.8	38.6 ± 4.2	47.56 ± 18.57	34.3 ± 10.6

a Measured on the day of sacrifice.

b Measured in blood plasma prepared on the day of animal sacrifice.

T-CHO: total cholesterol; TG: triglyceride; FBG: fasting blood glucose; HDL: high-density lipoprotein; LDL: low-density lipoprotein; SA: serum albumin (g/L); AT: alanine transporter (U/L). 2 M: normal 2-month-old mice, 12 M, 12 M + Vehicle and 12 M + Naringenin, 12 M + Vehicle: 0.5% CMC-Na treated 12-month-old mice, 12 M + Naringenin: Naringenin (100 mg/kg/day) treated 12-months-old mice.

One-way ANOVA, post hoc Tukey’s test (**p < 0.01 compared to the 2 M group, ^#^ *p* < 0.05 compared to the 12 M + vehicle group).

### 3.3 Protective effect of naringenin on aging-induced retinal histological changes in mice

**FIGURE 2:**
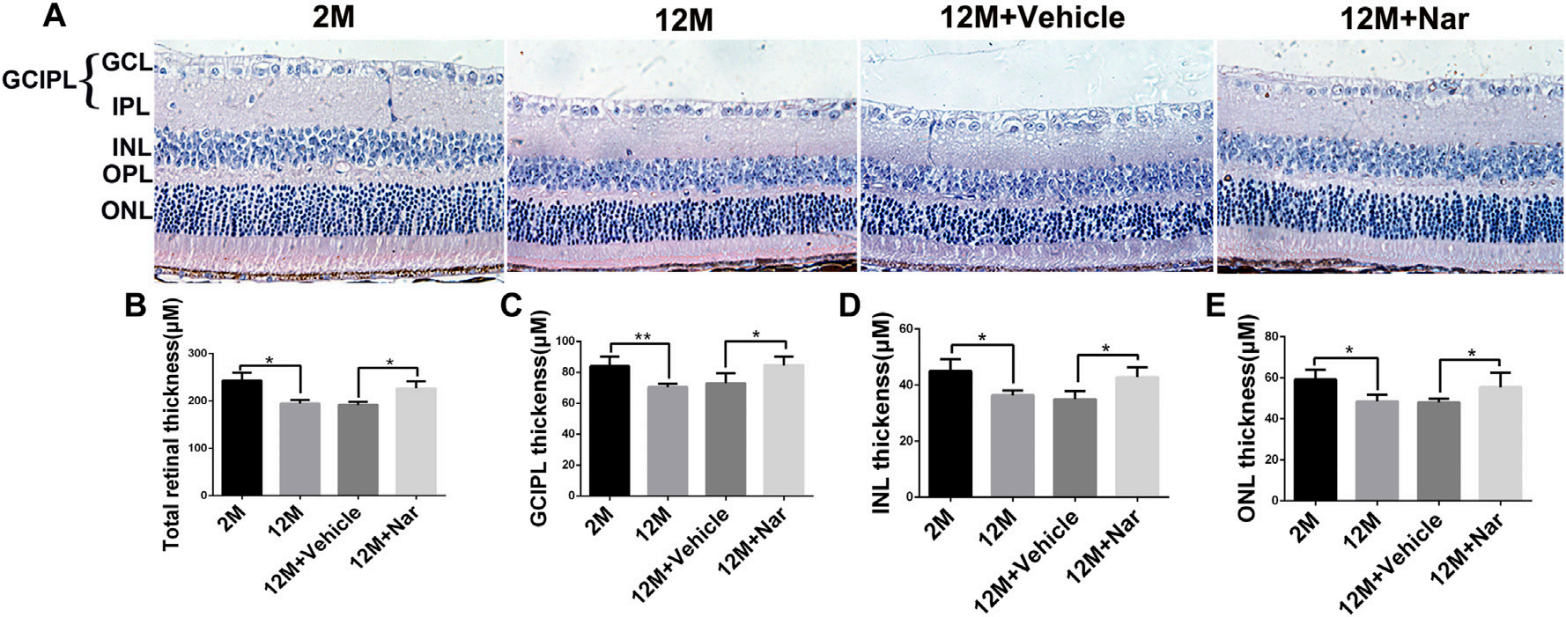
Naringenin prevented aging-induced retinal degeneration. **(A)** Hematoxylin and eosin staining was observed using an inverted fluorescence microscope from 2 M, 12 M, 12 M + Vehicle, and 12 M + Naringenin. Magnification 40×. **(B)** The retinal thickness of the total retina. **(C)** The thickness of the GCIPL. **(D)** The thickness of the INL. **(E)** The thickness of the ONL. GCIPL, ganglion cell and inner plexiform layer; INL, inner nuclear layer; OPL, outer plexiform layer; ONL, outer nuclear layer. The results are expressed as the mean values ± SEM, *n* = 5. One-way ANOVA, post hoc Tukey’s test (*p < 0.05, **p < 0.01).

Retinal thickness decreases with aging, and this change may be related to decreased retinal function (Nadal-Nicolás et al., 2018). H&E staining was used to evaluate retinal degeneration at different ages. We observed that the administration of naringenin prevented this reduction in retinal thickness in the total retina, ganglion cell, inner plexiform layer (GCIPL), inner nuclear layer (INL), and outer nuclear layer (ONL) (*p < 0.05, **p < 0.01; Figure 2).

### 3.4 Naringenin improved the abnormal mitochondrial dynamics

**FIGURE 3:**
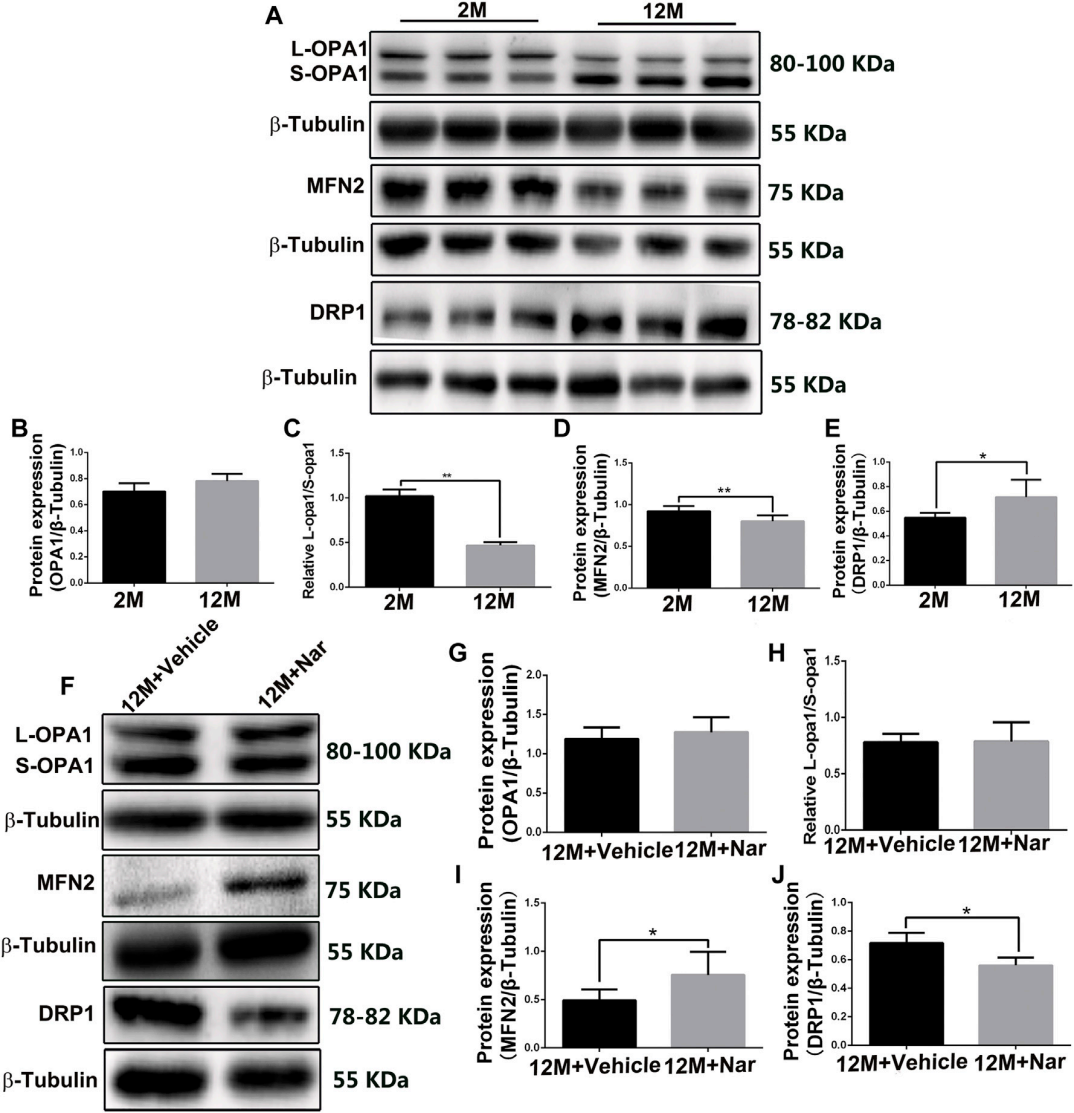
Naringenin promoted mitochondrial fusion and decreased mitochondrial fragmentation in aging mice. **(A)** Representative western blot of OPA1, MFN2, and DRP1 at different ages. Graphic representation of average fold changes in **(B)** OPA1, **(C)** L-OPA1/S-OPA1, **(D)** MFN2, and **(E)** DRP1 protein expression. **(F)** Representative western blot of OPA1, MFN2, and DRP1 in treatment with naringenin, Graphic representation of average fold changes in **(G)** OPA1, **(H)** L-OPA1/S-OPA1, **(I)** MFN2, and **(J)** DRP1 protein expression. The results are expressed as the mean values ± SEM, *n* = 5. Unpaired Student’s t-test (*p < 0.05, **p < 0.01).

In the physiologically aging retina, we observed that the ratio of L-OPA1/S-OPA1 and MFN2 protein levels significantly decreased, while that of the mitochondrial fission protein DRP1 was increased (*p < 0.05, **p < 0.01; Figures 3A–E), indicating that mitochondria tend to fragment with aging. The abnormal mitochondrial dynamics were improved by treatment with naringenin, and expression of the mitochondrial fusion regulator protein MFN2 was increased; however, the total OPA1 proteins and the ratio of L-OPA1/S-OPA1 were not significantly altered, and mitochondrial fission of DRP1 proteins was decreased (*p < 0.05; Figures 3F–J).

### 3.5 Naringenin upregulated autophagy in aging mouse retina by adenosine monophosphate-activated protein kinase/rapamycin pathway

**FIGURE 4:**
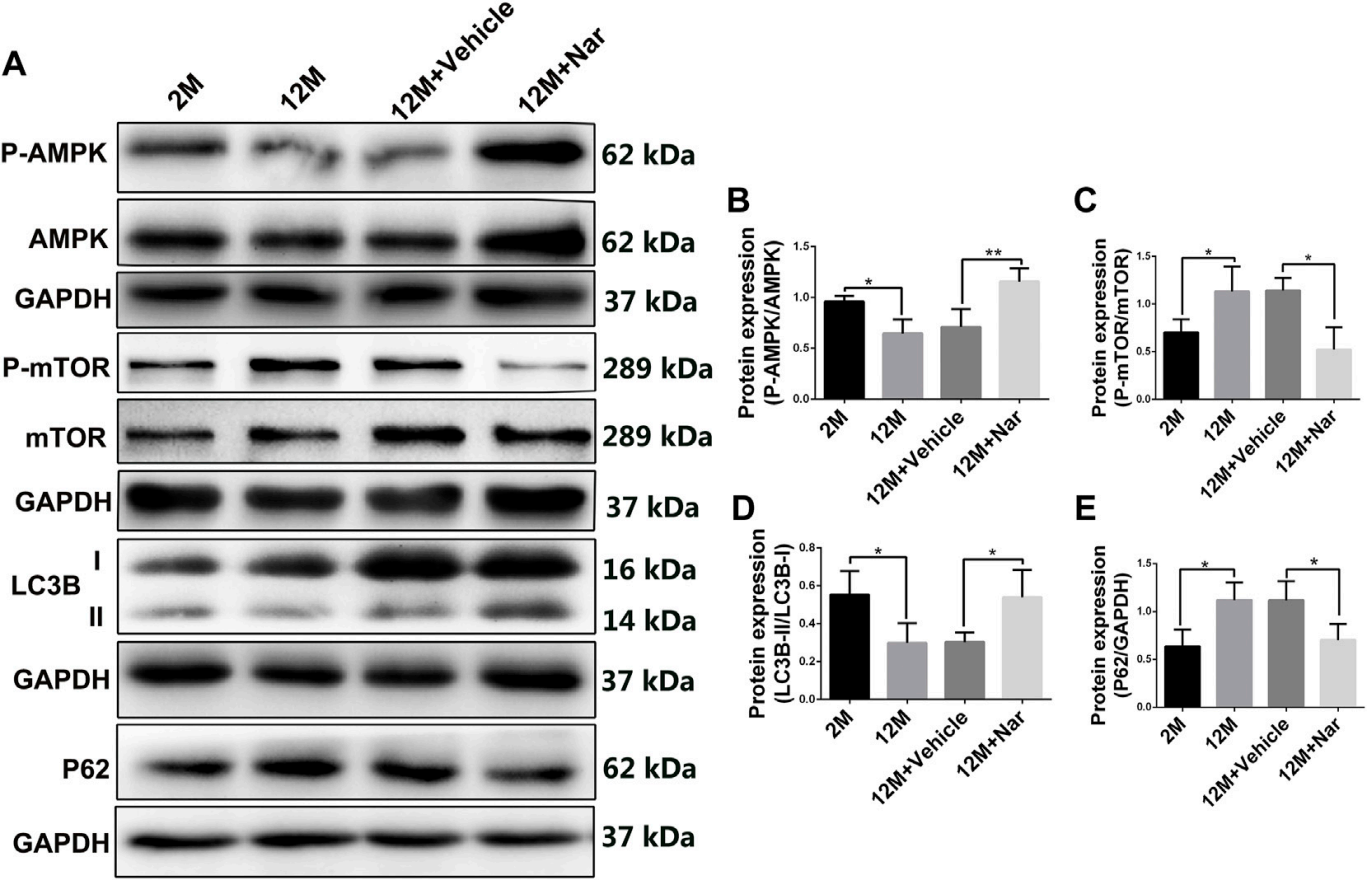
Naringenin upregulated autophagy in aging mouse retina via the AMPK/mTOR pathway. **(A)** Representative western blots of P-AMPK, AMPK, P-mTOR, mTOR, LC3B, and P62. **(B)** Relative expression levels of P-AMPK/AMPK protein. **(C)** Relative expression levels of P-mTOR/mTOR protein. **(D)** Relative expression levels of LC3B-II/I protein. **(E)** Relative expression levels of P62 protein. The results are expressed as the mean values ± SEM, *n* = 5. One-way ANOVA, post hoc Tukey’s test (*p < 0.05, **p < 0.01).

Autophagy is essential for clearing damaged or energy-deficient mitochondria, which otherwise accumulate and induce mitochondria-mediated cell death (Wohlgemuth et al., 2010). As shown in Figure 4, LC3B-II/I decreased while P62 increased in the retina of the 12-month-old mice compared with that of the 2-month-old mice. However, the downregulation of aging-related alterations in autophagy was significantly rescued by naringenin treatment (*p < 0.05; Figures 4A,D,E). Aging induced a significant decrease in P-AMPK levels; however, this effect was attenuated by treatment with naringenin. In addition, P-mTOR tended to be upregulated in the aging mice, whereas its expression was greatly reduced after treatment with naringenin (*p < 0.05; Figures 4A–C).

## 4 Discussion

Eye aging occurring in the retina is closely related to age-related eye diseases (Yang et al., 2019). There has been an annual increase in the incidence of age-related eye diseases with an increase in the aging population (Voleti and Hubschman, 2013). Aging-related eye diseases such as glaucoma and AMD have become major diseases that seriously threaten vision (Francisco et al., 2020). However, there is a lack of safe and effective dietary supplements to treat or delay aging in the eyes. In this study, we established a physiologically aging mouse model using long-term oral naringenin for 10 months to investigate potential anti-aging mechanisms. This study provides evidence that: 1) treatment with naringenin ameliorates retinal function in aging mice and aging-induced histological changes in the retina; 2) Naringenin promotes mitochondrial fusion and decreases mitochondrial fragmentation, and 3) Naringenin upregulates autophagy through the AMPK/mTOR pathway in the retina of the aging mice.

The ERG is a common and sensitive measurement used to evaluate retinal function (Tang et al., 2021). The a-wave provides information associated with the photoreceptors (cones and rods) and b-waves regarding the physiology of bipolar and Müller cells (Scalabrino et al., 2015). Small oscillations, called oscillatory potentials, can be observed on the leading edge of the b-wave and are thought to be derived from amacrine cells (Cécyre et al., 2020). In humans, aging is associated with decreased cone and rod responses in the ERG (Weleber, 1981). Our results showed that, long-term oral administration of naringenin partially preserved visual function that significantly attenuated aging-induced decreases in a- and b-waves amplitudes. Furthermore, treatment with naringenin counteracted aging-induced degeneration of retinal structure in various layers. Interestingly, Piano et al. reported that treatment with naringenin effectively prolonged the survival of cones, which generally die as a consequence of rod disappearance (Piano et al., 2019). The long-term oral administration of naringenin preserves retinal function by increasing photopic b-wave amplitude (Piano et al., 2021). These results indicate that naringenin protects against aging-induced degeneration in visual function by ensuring the survival of retinal cells, especially cones and rods.

MFN2 and OPA1 are important fusion regulators located in the mitochondrial outer and inner membranes. In contrast, DRP1 is an important fission regulator located in the mitochondrial outer membrane (Wai and Langer, 2016). The mitochondria tend to fragment with age, indicating reduced fusion and/or increased division (Liesa and Shirihai, 2013). Naringenin mediates protective effects against cardiac dysfunction by inhibiting mitochondrial damage and regulating the expression of OPA1 (Ye et al., 2020). In our study, we observed that MFN2 protein expression increased, OPA1 protein expression and the ratio of L-OPA1/S-OPA1 were unchanged, and DRP1 protein expression decreased in the 12-month-old mice treated with naringenin compared with the 12-month-old mice treated with vehicle, indicating that fission was decreased and fusion was increased.

Autophagy is a lysosomal degradation process that renews cellular components and plays an important role in cell homeostasis (Wang et al., 2015). Mitochondrial fragmentation is a prerequisite for autophagy and may be physiologically favorable (Twig et al., 2008). We further observed that autophagy flux markers, such as LC3 and P62, were activated in response to treatment with naringenin in the retina of the aging mice, suggesting that maintenance of mitochondrial dynamics balance may require autophagy coordination. AMPK promotes autophagy by phosphorylating autophagy-related proteins, including mTOR (Ye et al., 2020). The reduction of mTOR activity can promote mitochondrial fusion and activate mitophagy/autophagy, thereby delaying the aging process (Maiese, 2016). In our study, aging induced a significant decrease in P-AMPK; however, this effect was attenuated by treatment with naringenin. P-mTOR tended to be upregulated in the aging mice. In contrast, P-mTOR expression was greatly reduced after treatment with naringenin. These results suggest that naringenin promotes autophagy in the aging mouse retina *via* the AMPK/mTOR pathway.

Numerous dietary compounds, ubiquitous in fruits, vegetables and spices have been isolated. These compounds include flavonoid and non-flavonoid polyphenols. These plant products, such as curcumin, resveratrol, and flavonoids, have health promoting effects emerged because their intake was related to a reduced incidence of age-related diseases (Bisht et al., 2010). Our previous study suggested that resveratrol protects eyes from glaucomatous neurodegeneration (Luo et al., 2018). Naringenin is a flavonoid compound (Arinç et al., 2015). In this study, naringenin preserved retinal function and structure in the aging mice. Vitamin B_3_ is an essential nutrient supplement for humans. Vitamin B_3_ supplementation protects against glaucoma and potentially other age-related neurodegenerations (Williams et al., 2017). Naringenin is also a common ingredient in the human diet (Nouri et al., 2019). Our results suggest that long-term oral administration of naringenin counteracts aging-related retinal degeneration. Naringenin may be a potential dietary supplement for the prevention or treatment of age-related eye diseases.

This study had some limitations. First, since the metabolic process of naringenin *in vivo* is complex, we cannot exclude the possibility that the anti-aging properties of naringenin observed in mice is due to some active metabolites (Testai et al., 2020). Secondly, the expression level of the fission and fusion proteins indirectly reflect the balance of fission and fusion. Immunofluorescence labeling of mitochondria and confocal imaging would be encouraged to observe the fission and fusion. In addition, age between 2 and 12 months does not reflect the entire aging process in C57BL/6J mice. Ages between 12 and 24 months require further exploration. Finally, further investigations using other aging models should be performed to better understand the integrated pharmacological activities of naringenin.

In conclusion, naringenin preserved retinal function and structure in the aging mice. The underlying mechanism may be associated with the fact that naringenin improved mitochondrial dynamics and mediated the mTOR signaling pathway to promote autophagy initiation. Age-related eye diseases, such as AMD and glaucoma, are closely related to autophagy and mitochondrial dynamics (Baek et al., 2021; Skeie et al., 2021). Therefore, naringenin may be a promising candidate for treating age-related eye diseases *via* regulating autophagy and mitochondrial dynamics.

## Data Availability

The raw data supporting the conclusions of this article will be made available by the authors, without undue reservation.

## Abbreviations

AMPK, adenosine monophosphate-activated protein kinase; AMD, age-related macular degeneration; DRP1, dynamin-related protein 1; ERG, electroretinogram; GCIPL, ganglion cell and inner plexiform layer; H&E, hematoxylin and eosin; INL, inner nuclear layer; L-OPA1, long-optic atrophy 1; LC3, microtubule-associated protein 1 light chain 3; MFN1, mitochondrial fusion protein 1; MFN2, mitochondrial fusion protein 2; mTOR, rapamycin; OPA1, optic atrophy 1; ONL, outer nuclear layer; S-OPA1, short-optic atrophy 1.
