# Supplementary figures and images for: Long-term oral administration of naringenin counteracts aging-related retinal degeneration via regulation of mitochondrial dynamics and autophagy
Source: Front Pharmacol. 2022 Jul 14;13:919905. doi: 10.3389/fphar.2022.919905 (PMC9330024; doi:10.3389/fphar.2022.919905)

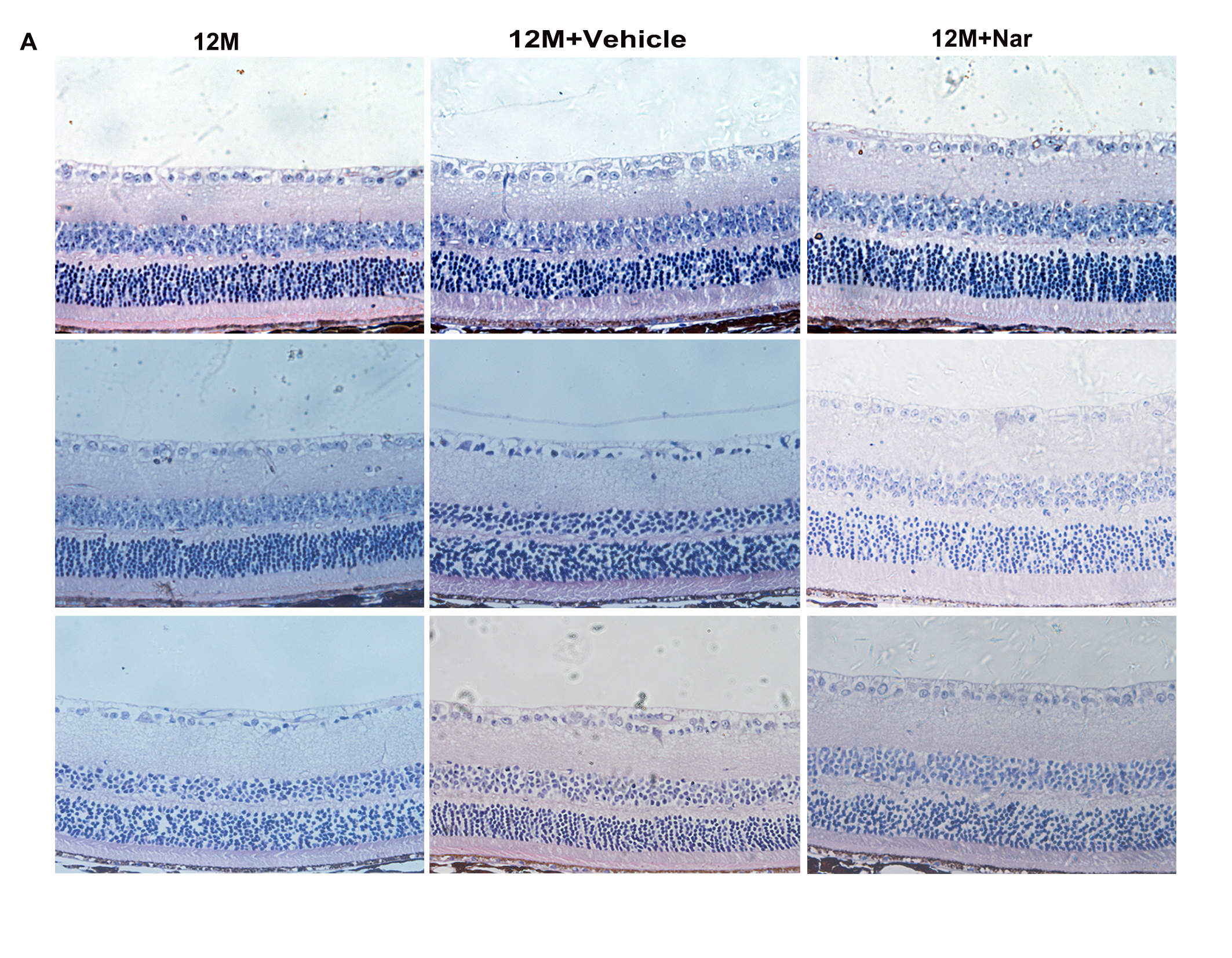

Supplement: Supplementary file 1 [file Image1.TIF]
